# Novel technique of performing transbronchial lung cryobiopsy (TBLC) for diagnosing diffuse parenchymal lung diseases (DPLD) in infants

**DOI:** 10.1002/rcr2.1096

**Published:** 2023-02-06

**Authors:** Tinku Joseph, Sumita Agrawal, Sreeraj Nair, Chandrasekhara Jayakumar, Rajesh Gopalakrishnan, Ajit Nambiar

**Affiliations:** ^1^ Division of Adult & Paediatric Interventional Pulmonology Amrita Institute of Medical Sciences Kochi India; ^2^ Department of Paediatrics Amrita Institute of Medical Sciences Kochi India; ^3^ Department of Cardiac Anaesthesia Amrita Institute of Medical Sciences Kochi India; ^4^ Department of Pathology Amrita Institute of Medical Sciences Kochi India

**Keywords:** diffuse parenchymal lung diseases, interventional pulmonology, paediatric rigid bronchoscopy, transbronchial lung cryobiopsy

## Abstract

Childhood interstitial lung disease (ChILD) diagnosis often requires surgical lung biopsy after the common causes are ruled out. However, surgical lung biopsy has its own set of complications. Hence transbronchial lung cryobiopsy has been used in various studies of adult interstitial lung disease (ILD) with good yield and minimal complications. But this newer mode is rarely used in diagnosing children with suspected ILD. Here, we present the first case of the use of this technique in an infant via a rigid tracheoscope.

## Graphical abstract

Transbronchial lung cryobiopsy (TBLC) has been reported to have high diagnostic yield for adult interstitial lung disease (ILD) with limited complications. However, this technique has not been utilised for diagnosis of diffuse parenchymal lung diseases (DPLD) in an infant. We present the first case of infant lung cryobiopsy done using rigid tracheoscope in a suspected case of childhood interstitial lung disease.

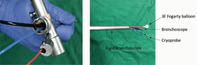

## INTRODUCTION

Despite multiple studies, the method of diagnosing diffuse parenchymal lung diseases (DPLDs) remains debatable, due to the merits and demerits of various procedures. Although surgical lung biopsy remains the gold standard due to its high yield, the associated morbidity and mortality limits its utilization.^1^, ^2^ Transbronchial lung cryobiopsy (TBLC) has been used in various studies for the diagnosis of ILD in adults with diagnostic yield (DY) ranging from 83% to 85% and limited complications.^1^, ^3^

This technique, however, has never been utilized for diagnosis in the infant population. We hereby present the first case in the world where this novel technique was used for the diagnosis of DPLD in an infant.

## CASE REPORT

An 11‐month‐old infant suffered from a recurrent history of cough and breathlessness since birth, for which he had been receiving treatment for at multiple hospitals. In view of the non‐responsiveness to the treatment given, a computed tomography (CT) of the chest was done which was suggestive of a probable diffuse parenchymal lung disease. For further management, including a lung biopsy for histopathological confirmation, the child was referred to our hospital. Upon arrival in the emergency department, he presented with an intermittent fever which had been ongoing for the preceding 2 weeks, was tachypneic, and had an oxygen saturation in room air of 92%. He was admitted to the intensive care unit and initiated on intravenous empirical antibiotics and supportive measures.

**FIGURE 1:**
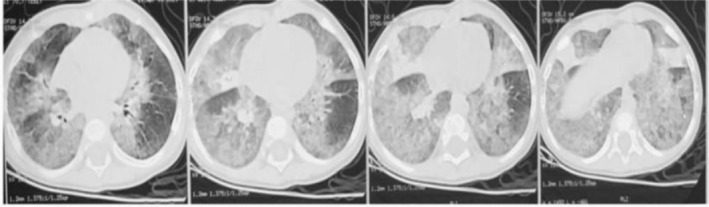
CT Chest: Bilateral diffuse ground glass opacities involving entire lung fields

He was extensively investigated for the cause of the illness. The chest X‐ray showed diffuse reticulonodular opacities in bilateral lower zones and a CT chest showed bilateral diffuse ground glass opacities with consolidatory patches in the lower lobes (Figure 1). Laboratory investigations included: Haemoglobin: 10.4 g/dl; total leucocyte count: 19,960/cumm; platelets: 453,000/cumm; C‐reactive protein: 68 mg/l. Blood culture had no growth after incubation for 5 days and gastric aspirate for acid‐fast bacilli (AFB) was negative. The bronchoalveolar lavage (BAL) was also negative for Periodic acid‐Schiff stain, AFB smear, and GeneXpert. BAL cytology showed lymphocytic predominance, with no growth on bacterial, fungal, or mycobacterium culture. An echocardiography was also done to rule out congenital cardiac anomalies and it was normal.

A multidisciplinary team meeting involving a paediatrician, pulmonologist, and cardiothoracic surgeons discussed the case, and it was decided that a lung biopsy would be performed for diagnosis. Options of surgical or cryo lung biopsy were also discussed with the members and patients' family. Finally, the decision was made to do TBLC under general anaesthesia.

**FIGURE 2:**
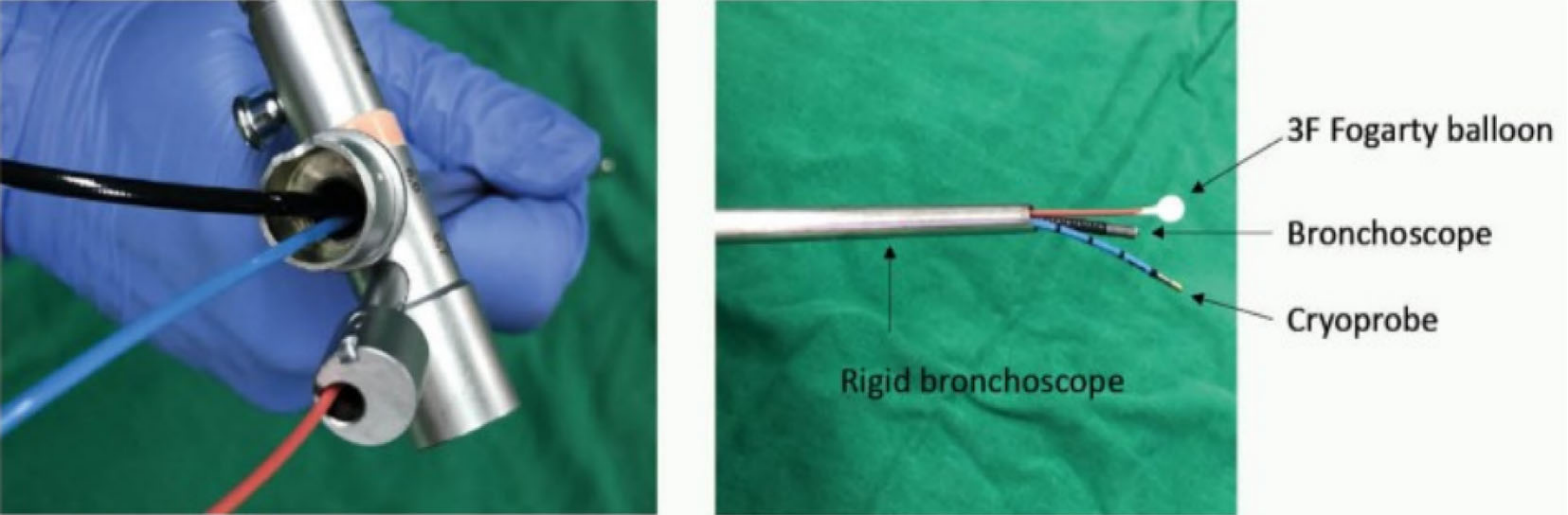
Modified transbronchial lung cryobiopsy technique used in this case

After obtaining written informed high risk procedural consent, the child was intubated with a 4 mm size rigid bronchoscope and airway was secured. Lung cryobiopsy was performed using a novel technique in which a 1.9 mm flexible cryoprobe (ERBE, Tubingen, Germany) was passed through the working channel of the rigid bronchoscope under fluoroscopic guidance into left lower lobe basal segments. To obtain post‐procedural haemostasis, a 2F Fogarty balloon was kept in the adjacent segment with the help of an ultrathin flexible bronchoscope (Olympus BF XP 190, Japan). Multiple transbronchial lung cryobiopsies were taken from the left basal segments (Figure 2). Adequate haemostasis was achieved post procedure and there was no evidence of pneumothorax on the fluoroscopic image taken immediately. The child was also extubated on the table after the procedure. There were no immediate or delayed post‐procedure complications seen.

**FIGURE 3:**
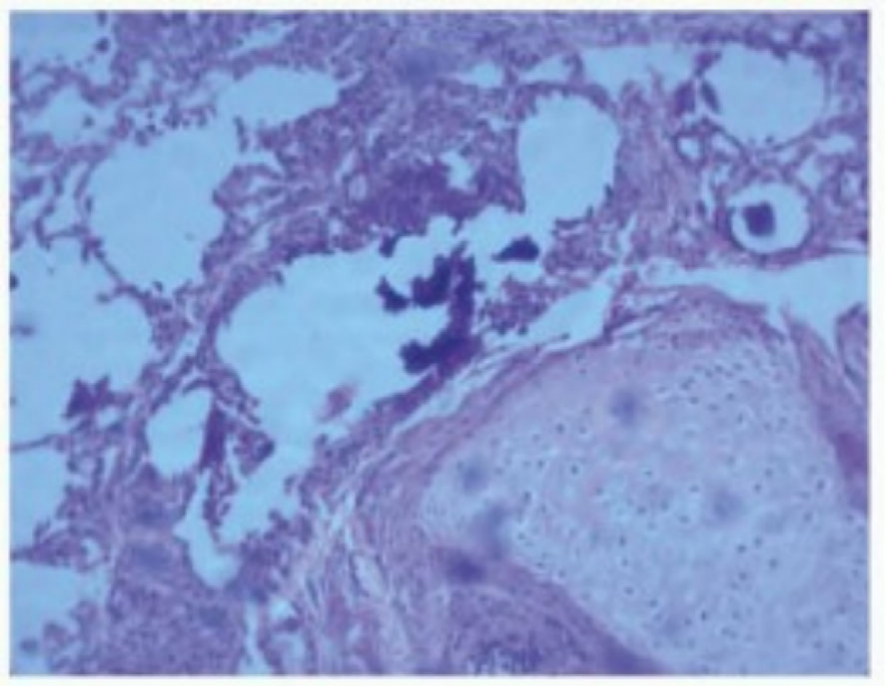
Histopathology image of the transbronchial lung biopsy specimen: Periodic acid–Schiff‐positive multiple microliths within the alveolar lumen

Histopathological examination of the transbronchial lung biopsy specimen showed lamellated concretions in the alveolar lumina suggestive of alveolar microlithiasis (Figure 3). Genetic mutation analysis of the SLC34A2 gene showed exonic deletion, classified as a pathogenic variant that contributes to pulmonary alveolar microlithiasis.

In view of the progressive nature of the illness, guarded prognosis, and no definitive medical treatment available, supportive care was given to the child. He was treated with systemic corticosteroids, calcium‐chelating molecules and oxygen supports. Despite our best efforts, the child continued to have repeated lower respiratory tract infections and succumbed to his illness after a month.

## DISCUSSION

Although surgical lung biopsy remains the gold standard for diagnosis of DPLD, it is associated with high mortality (2%–6%) at 90 days and increased risk of complications including pneumothorax, bronchopleural fistula, re‐surgery, prolonged hospitalization, and persistent pain.^1^, ^2^ Transbronchial forceps biopsy is less invasive but gives limited yield. Since the advent of transbronchial lung cryobiopsy, it has been investigated for diagnosis of ILD and has given good yield with minimal complications.^2^, ^3^

A study by Ravaglia et al compared the role of TBLC versus surgical lung biopsy (SLB) and showed that the DY for TBLC was 82.8% versus 98.7% in surgical lung biopsy. However, the mortality was 0.3% in the TBLC group compared to 2.7% in SLB group. Also, the median time of hospitalization was 2.6 days in TBLC and 6.1 days in SLB group. The most common complication in TBLC group was pneumothorax (20.2%). No significant bleeding was seen.^3^

The American Thoracic Society (ATS) guideline for chILD recommends a surgical lung biopsy for diagnosis after common causes of diffuse lung disease have been ruled out.^4^ Hence, after a detailed evaluation and multi‐disciplinary team meeting at our center, it was decided that this patient required a biopsy for further evaluation. We, hence, performed the first TBLC in an infant via the use of a 4 mm rigid tracheoscope with no immediate post‐procedure complications.

A similar attempt has been reported in a 10‐year‐old boy by Srikanta et al, with no immediate post‐procedure complications.^5^ Another observational study of 28 paediatric patients ranging in age from 22 months to 17 years who underwent TBLC reported a DY of 92.8%. This study reported minor complications including mild bleeding, transient hypoxemia, and bronchospasm.^6^

In conclusion, since TBLC is less invasive and associated with minimal complications, this diagnostic modality can be used in the paediatric population for ILDs. Further studies, however, are needed to understand the safety and efficacy of the procedure, especially in the infant population.

## CONFLICT OF INTEREST STATEMENT

None declared.

## ETHICS STATEMENT

The authors declare that appropriate written consent was obtained for the publication of this manuscript and accompanying images.

## Data Availability

Data available on request due to privacy/ethical restrictions.

## References

[1] Colella S , Haentschel M , Shah P , Poletti V , Hetzel J . Transbronchial lung cryobiopsy in interstitial lung diseases: best practice. Respiration. 2018;95(6):383–91. 29894993 10.1159/000488910

[2] Raghu G , Remy‐Jardin M , Myers JL , Richeldi L , et al. Diagnosis of idiopathic pulmonary fibrosis. An official ATS/ERS/JRS/ALAT clinical practice guideline. Am J Respir Crit Care Med. 2018;198(5):e44–68. 30168753 10.1164/rccm.201807-1255ST

[3] Ravaglia C , Bonifazi M , Wells AU , Tomassetti S , Gurioli C , Piciucchi S , et al. Safety and diagnostic yield of transbronchial lung cryobiopsy in diffuse parenchymal lung diseases: a comparative study versus video‐assisted thoracoscopic lung biopsy and a systematic review of the literature. Respiration. 2016;91(3):215–27. 26926876 10.1159/000444089

[4] Kurland G , Deterding RR , Hagood JS , Young LR , Brody AS , Castile RG , et al. An official American Thoracic Society clinical practice guideline: classification, evaluation, and management of childhood interstitial lung disease in infancy. Am J Respir Crit Care Med. 2013;188(3):376–94. 23905526 10.1164/rccm.201305-0923ST PMC3778735

[5] Srikanta JT , Swarna S , Shylendra DS , Mehta R . Transbronchial lung cryobiopsy for diagnosis of pediatric interstitial lung disease. Indian Pediatr. 2018;55(6):519–20. 29978822

[6] Moslehi MA . Transbronchial lung Cryobiopsy in children. Expert Rev Respir Med. 2022;16(3):333–9. 10.1080/17476348.2021.1987884 34602011

